# Does intraoperative cyst rupture of malignant cystic renal masses really have no negative impact on oncologic outcomes?

**DOI:** 10.1186/s12957-022-02824-7

**Published:** 2022-11-25

**Authors:** Peirong Xu, Sihong Zhang, Bohong Cao, Jiaqi Huang, Yaohui Li, Jiangting Cheng, Wenyao Lin, Jie Cheng, Weijie Chen, Yanjun Zhu, Shuai Jiang, Xiaoyi Hu, Jianming Guo, Hang Wang

**Affiliations:** 1 Department of Urology, Xuhui Hospital, Fudan University, 966th Huaihai Middle Rd, Xuhui District, Shanghai, China; 2 Department of Radiology, Zhongshan Hospital, Fudan University, 180th Fengling Rd, Xuhui District, Shanghai, China; 3 Department of Urology, Minhang Hospital, Fudan University, 170th Xinsong Rd, Minhang District, Shanghai, China; 4 Department of Urology, Zhongshan Hospital, Fudan University, 180th Fengling Rd, Xuhui District, Shanghai, 200032 China; 5 Department of Pathology, Zhongshan Hospital, Fudan University, 180th Fengling Rd, Xuhui District, Shanghai, China

**Keywords:** Cystic kidney diseases, Renal cell carcinoma, Nephrectomy, Rupture, Classification

## Abstract

**Background:**

To assess the impact of malignant cystic renal masses (CRM) rupture on oncologic outcomes.

**Methods:**

The study included 406 cases with partial nephrectomy (PN) and 17 cases with cyst decortication confirmed as malignant CRM by pathology. Recurrence-free survival (RFS), metastasis-free survival (MFS), cancer-specific survival (CSS), and overall survival (OS) were analyzed by the Kaplan-Meier method and log-rank test. Cox regression was used to identify risk factors associated with RFS, MFS, CSS, and OS. Logistic regression was performed to explore predictors of rupture.

**Results:**

Tumor rupture occurred in 32 of 406 cases (7.9%). With median follow-up of 43 months, 4 (12.5%) and 5 (1.3%) cases experienced recurrence in rupture and non-rupture group, respectively (*P* = 0.003). Estimated RFS, MFS, and CSS were shorter in cyst ruptured (CR) group than non-ruptured (nonCR) cases (*P* < 0.001; *P* = 0.001; *P* < 0.001). Cox regression analysis indicated that CR was an independent prognostic factor for RFS (*HR* = 7.354; 95% *CI* = 1.839–29.413; *P* = 0.005), MFS (*HR* = 8.069; 95% *CI* = 1.804–36.095; *P* = 0.006), and CSS (*HR* = 9.643; 95% *CI* = 2.183–42.599; *P* = 0.003). Multivariable logistic regression showed that Bosniak IV was a protective factor for CR (*OR* = 0.065; 95% *CI* = 0.018–0.239; *P* < 0.001). However, compared to Bosniak III and I-IIF, Bosniak IV CRMs showed higher rate of clear cell renal cell carcinoma (ccRCC) (76.8% vs 36.5% vs 81.4%) (*P* < 0.001) and lower rate of Fuhrman I staging (11.2% vs 66.7% vs 7.4%) (*P* < 0.001). Therefore, in ruptured cases, the recurrence rate was higher in CRM with Bosniak IV (50%, 2/4) than Bosniak I-III (4.4%, 2/45) (*P* = 0.029).

**Conclusions:**

Intraoperative malignant CRM rupture had negative impacts on oncologic outcomes. Bosniak IV was more aggressive than Bosniak I-III and had a higher risk of recurrence after rupture. However, Bosniak IV had a lower risk of rupture, which could weaken even cover-up of the true effect of tumor rupture on oncologic outcomes.

## Introduction

The incidence of cystic renal masses (CRM) has increased rapidly over the past few decades due to the widespread use of cross-sectional imaging [1]. Partial nephrectomy (PN) has been established as a standard treatment for small renal masses [2, 3] as it maintains similar oncologic outcomes with radical nephrectomy [4, 5] and meanwhile preserves renal function [6, 7]. Especially, off-clamp PN offers a superior renal function preservation [8–10]. When CRM rupture occurs during PN procedure, it is disconcerting for the surgeon because of a theoretical risk of tumor recurrence [11–13]. However, Pradere B. et al. [14] recently reported that intraoperative cyst rupture (CR) at PN of CRM did not increase the risk of recurrence. Although encouraging, this study has some limitations including small sample size, various surgical and clinical experiences, 25% benign CRM rupture without clinical significance, lack of pathological stratification, and the short follow-up time of CR. Therefore, the larger population-based study with 406 patients pathologically diagnosed as malignant CRM in our institution was conducted to externally discover the relationship between intraoperative CR and tumor recurrence.

## Patients and methods

### Study population

**Fig. 1:**
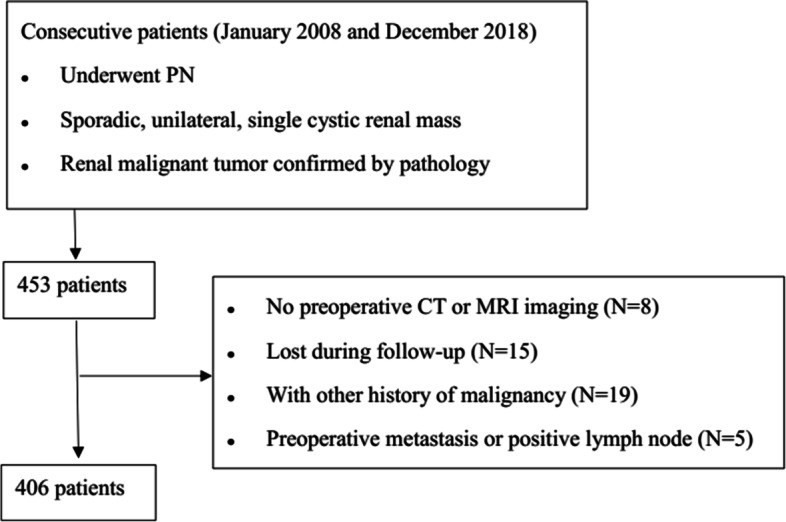
The flow-chart image of patients selection

With the approval from institutional review board, we retrospectively reviewed 406 patients including 106 females and 300 males, who underwent PN for the CRM and were confirmed as malignant tumor by postoperative pathology at our center between January 2008 and December 2018. The inclusion criteria and exclusion criteria are shown in Fig. 1. All PNs were performed by 4 experienced surgeons with more than 50 procedures. The Bosniak classification and RENAL nephrometry score were evaluated based on contrast-enhanced computerized tomography scan and/or magnetic resonance imaging. Electronic medical records were retrospectively reviewed to identify personal characteristics in which the surgery records were all carefully reviewed and any description of rupture, effraction, puncture, and/or content leakage of the cyst masses was considered as CR, which was consistent with the previous study [14]. Another 17 cases with cyst decortication (CD) confirmed as malignant tumors by postoperative pathology in our institution were also analyzed. CD was performed to patients preoperatively diagnosed as benign CRM with only removal of the cover of renal cyst protruding from kidney surface. Within all the patients who underwent CD, 17 were confirmed as malignancy by final pathology and taken into account considering that the entity of CRM was destroyed. Tumor recurrence was defined as a new lesion in the resection bed, regional lymph nodes, or distant organs metastasis after surgery, which was also consistent with the previous study [14].

### Surgery

Open partial nephrectomy included traditional open partial nephrectomy and mini-incision open partial nephrectomy, as previously described in detail [15]. Minimally invasive partial nephrectomy (MIPN) included laparoscopic partial nephrectomy and robot-assisted partial nephrectomy [16]. The da Vinci surgical system (Intuitive Surgical, Inc., Sunnyvale, CA, USA) was used in robot-assisted partial nephrectomy (RAPN). The tumor was excised with a small surrounding margin of normal renal parenchyma. After excision of the tumor, all transected blood vessels on the tumor resection bed were exactly stitched with 3-0 Vicryl sutures. The residual renal parenchyma was closed with 2-0 Vicryl sutures. Finally, adjunct hemostatic agents were used. All these CR cases were irrigated with large amounts of distilled water.

### Statistical analysis

Data were analyzed with SPSS software version 21.0 (IBM SPSS). The independent sample *t*-test was used to compare quantitative variables, and the chi-square test or Fisher’s exact test was used to compare qualitative variables. Recurrence-free survival (RFS), metastasis-free survival (MFS), cancer-specific survival (CSS), and overall survival (OS) were analyzed by the Kaplan-Meier method and log-rank test. Cox proportional hazards regressions were used to identify risk factors associated with RFS, MFS, CSS, and OS. Univariate and multivariable logistic regressions were performed to determine predictors of CR. All tests were two sides, and statistical significance was considered at *P* < 0.05.

## Results

### Oncologic outcomes of intraoperative CR

**Fig. 2:**
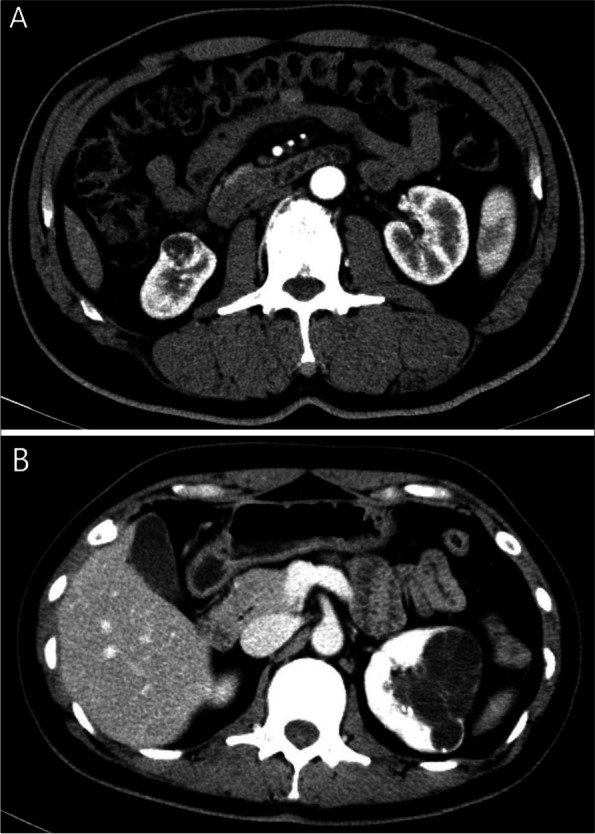
The representative imaging of CRM with nonCR (**A**) and CR (**B**)

**Fig. 3:**
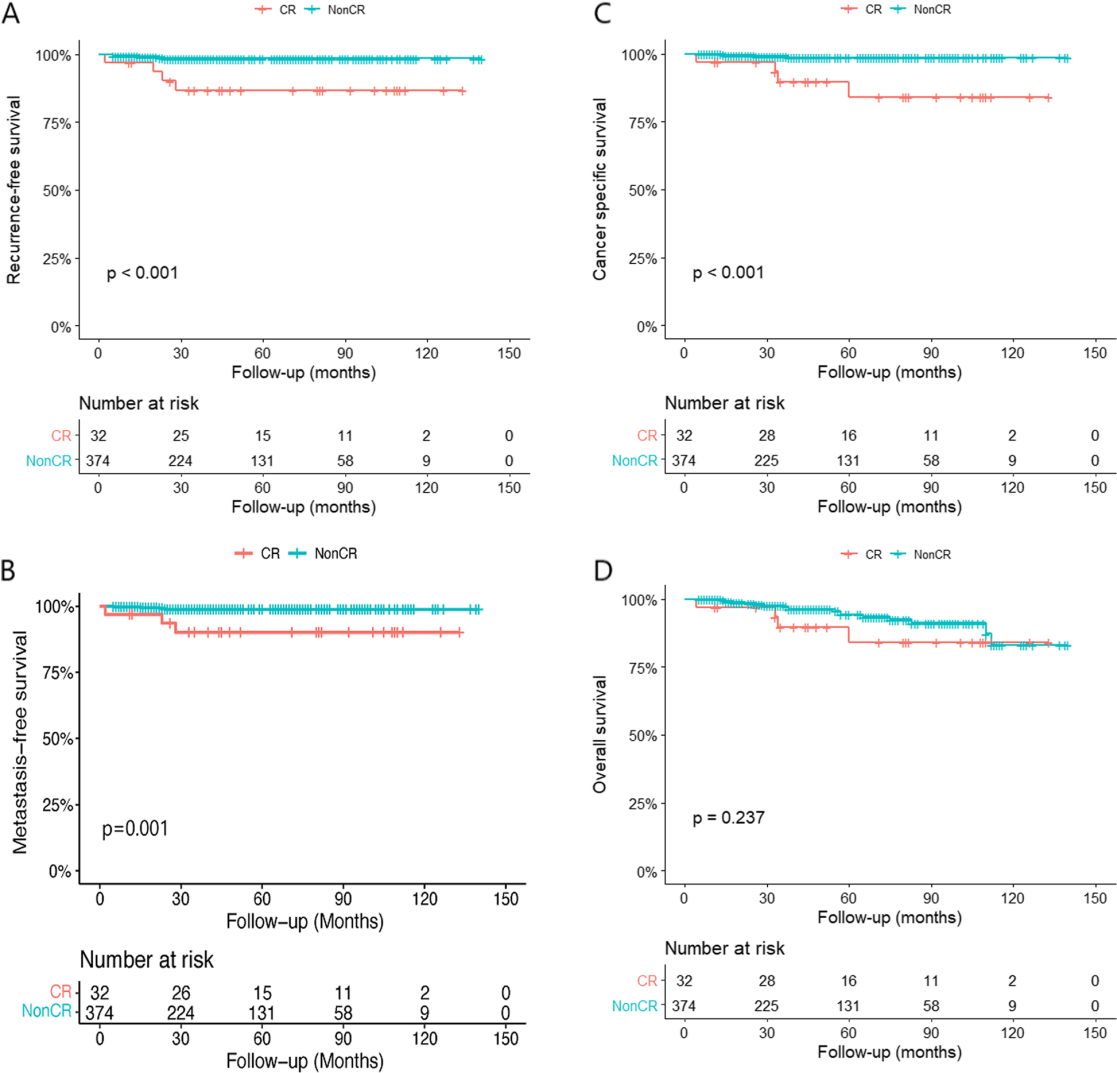
The recurrence-free survival (**A**), metastasis-free survival (**B**), cancer-specific survival (**C**), and overall survival (**D**) between patients with cyst rupture (CR) and without CR

Of 406 patients who underwent PN, CR occurred in 32 cases (7.9%). The representative imaging of CRM with nonCR and CR has been supplied in Fig. 2. All surgical margin tests were negative. The median follow-up time was 43 (range 4 to 140) months for the whole cohort, 56 (range 4 to 133) months in CR group, and 42 (range 5 to 140) months in nonCR group, respectively. Five patients (1.3%) in nonCR group experienced recurrence at a median time of 15 months. However, 4 patients (12.5%) in CR group experienced recurrence at a median time of 21.5 months (*P* = 0.003). Comparison of demographic information and clinical data in patients with and without CR was presented in Table 1. Estimated RFS, MFS, and CSS of CR group were shorter than nonCR group (*P* < 0.001; *P* = 0.001; *P* < 0.001) (Fig 3 A, B, and C). Estimated OS did not differ significantly between patients with or without CR (*P* = 0.237) (Fig. 3D). The Cox regression analysis indicated that CR was an independent prognostic factor for RFS (*HR* = 7.354; 95% *CI* = 1.839–29.413; *P* = 0.005), MFS (*HR* = 8.069; 95% *CI* = 1.804–36.095; *P* = 0.006), CSS (*HR* = 9.643; 95% *CI* = 2.183–42.599; *P* = 0.003), but not OS (*HR* = 1.905; 95% *CI* = 0.642–5.654; *P* = 0.245) (Tables 2, 3, 4, and 5).

**Table 1:** Comparison of clinical characteristics in patients with cyst rupture (CR) and without CR

Variables	nonCR	CR	*p*-value
No. of patients	374	32	
No. of recurrence (%)	5 (1.3)	4 (12.5)	**0.003**
Mean ± SD age (range) (years)	52.94 ± 13.37 (15–85)	54.91 ± 12.78 (30–83)	0.423
Mean ± SD BMI (range) (kg/m^2^)	24.61 ± 3.40 (16.2–36.2)	23.92 ± 3.18 (18.8–29.8)	0.273
Mean ± SD RENAL score	7.18 ± 1.66 (4–11)	7.28 ± 1.63 (4–10)	0.752
Mean ± SD tumor size (cm)	2.94 ± 1.21 (1.0–8.0)	3.42 ± 1.77 (1.5–8.0)	0.139
No. of hypertension (%)	120 (32.1)	9 (28.1)	0.664
No. of diabetes (%)	55 (14.7)	8 (25.0)	0.147
No. of smoking (%)	67 (17.9)	7 (21.9)	0.578
Mean ± SD eGFR (range) (ml/min)	104.84 ± 11.3 (73–119)	101.85 ± 10.31 (75–118)	0.147
No. gender (%)			0.490
Male	278 (74.3)	22 (68.8)	
Female	96 (25.7)	10 (31.3)	
No. of tumor location (%)			0.107
Left kidney	184 (49.2)	11 (34.4)	
Right kidney	190 (50.8)	21 (65.6)	
No. of Bosniak classification (%)			**< 0.001**
IIF	27 (7.2)	8 (25.0)	
III	174 (46.5)	20 (62.5)	
IV	173 (46.3)	4 (12.5)	
No. of surgical approach (%)			0.119
OPN	216 (57.8)	23 (71.9)	
MIPN	158 (42.2)	9 (28.1)	
No. of Fuhrman classification (%)^a^			0.258
I	40 (12.1)	5 (23.8)	
II	272 (82.2)	15 (71.4)	
III	19 (5.7)	1 (4.8)	
IV	0	0	
No. of pathological type (%)			**< 0.001**
ccRCC	298 (79.7)	14 (43.8)	
MCRNLMP	36 (9.6)	6 (18.8)	
pRCC	15 (4)	5 (15.6)	
cRCC	12 (3.2)	2 (6.3)	
others^b^	13 (3.5)	5 (15.6)	
No. of malignant potential (%)^c^			**0.001**
High aggressiveness	304 (81.3)	18 (56.3)	
Low aggressiveness	70 (18.7)	14 (43.8)	

*BMI* body mass index, *OPN* open partial nephrectomy, *MIPN* minimally invasive partial nephrectomy, *ccRCC* clear cell renal cell carcinoma, *MCRNLMP* multilocular cystic renal neoplasm of low malignant potential, *pRCC* papillary renal cell carcinoma, *cRCC* chromophobe renal cell carcinoma

^a^ccRCC, pRCC, cRCC, RCC, unclassified and clear cell papillary renal cell carcinomas were graded by Fuhrman classification

^b^Others include MiT family translocation RCC, RCC, unclassified, mixed epithelial and stromal tumor, clear cell papillary renal cell carcinomas, thyroid-like follicular RCC, metastatic tumor, and renal carcinosarcoma

^c^ccRCC, type II pRCC and renal carcinosarcoma were classified as high aggressiveness, and other pathologies were classified as low aggressiveness

**Table 2:** The univariate and multivariate Cox regressions for RFS

Variables	Univariate analysis		Multivariate analysis	
	HR (95% *CI*)	*p*-value	*OR* (95% *CI*)	*p*-value
Age	0.996 (0.949–1.045)	0.861		
BMI	0.899 (0.736–1.098)	0.296		
RENAL score	1.203 (0.809–1.790)	0.361		
Tumor size	1.490 (0.993–2.235)	0.054	0.976 (0.621–1.532)	0.915
Gender				
Male				
Female	0.825 (1.171–3.973)	0.811		
Tumor location				
Left kidney				
Right kidney	1.180 (0.317–4.396)	0.805		
CR				
Nonrupture				
Rupture	8.841 (2.372–32.945)	**0.001**	7.354 (1.839–29.413)	**0.005**
Malignant potential				
Low aggressiveness				
High aggressiveness	2.076 (0.260–16.599)	0.491		
Pathological stage				
≤ I stage				
> II stage	15.902 (3.278–77.147)	**0.001**	16.457 (1.775–152.569)	**0.014**
Surgical approach				
OPN				
MIPN	0.203 (0.025–1.622)	0.132	0.241 (0.030–1.969)	0.184

**Table 3:** The univariate and multivariate Cox regressions for MFS

Variables	Univariate analysis		Multivariate analysis	
	HR (95% *CI*)	*p*-value	OR (95% *CI*)	*p*-value
Age	1.016 (0.959–1.076)	0.597		
BMI	0.920 (0.734–1.152)	0.465		
RENAL score	1.101 (0.700–1.730)	0.678		
Tumor size	1.000 (0.547–1.826)	0.999		
Gender				
Male				
Female	1.160 (0.225–5.978)	0.859		
Tumor location				
Left kidney				
Right kidney	1.261 (0.282–5.635)	0.762		
CR				
Nonrupture				
Rupture	8.069 (1.804–36.095)	**0.006**	7.310 (1.608–33.232)	**0.010**
Malignant potential				
Low aggressiveness				
High aggressiveness	1.556 (0.187–12.923)	0.683		
Pathological stage				
≤ I stage				
> II stage	8.629 (1.035–71.941)	**0.016**	6.296 (1.738–53.709)	**0.023**
Surgical approach				
OPN				
MIPN	0.023 (0.001–13.197)	0.244

**Table 4:** The univariate and multivariate Cox regressions for CSS

Variables	Univariate analysis		Multivariate analysis	
	HR (95% *CI*)	*p*-value	OR (95% *CI*)	*p*-value
Age	1.017 (0.963–1.073)	0.547		
BMI	0.875 (0.709–1.078)	0.210		
RENAL score	1.311 (0.845–2.032)	0.227		
Tumor size	1.419 (0.907–2.220)	0.125	0.807 (0.501–1.301)	0.380
Gender				
Male				
Female	0.931 (0.188–4.612)	0.930		
Tumor location				
Left kidney				
Right kidney	1.707 (0.407–7.150)	0.464		
CR				
Nonrupture				
Rupture	9.514 (2.369–38.215)	**0.001**	9.643 (2.183–42.599)	**0.003**
Malignant potential				
Low aggressiveness				
High aggressiveness	1.902 (0.234–15.468)	0.548		
Pathological stage				
≤ I stage				
> II stage	32.125 (5.851–176.382)	**< 0.001**	50.831 (4.579–564.296)	**0.001**
Surgical approach				
OPN				
MIPN	0.025 (0.001–11.055)	0.235

**Table 5:** The univariate and multivariate Cox regressions for OS

Variables	Univariate analysis		Multivariate analysis	
	HR (95% *CI*)	*p*-value	OR (95% *CI*)	*p*-value
Age	1.009 (0.978–1.041)	0.581		
BMI	0.936 (0.825–1.062)	0.305		
RENAL score	0.980 (0.735–1.306)	0.891		
Tumor size	1.007 (0.709–1.432)	0.967		
Gender				
Male				
Female	0.818 (0.302–2.219)	0.694		
Tumor location				
Left kidney				
Right kidney	1.858 (0.779–4.434)	0.162	1.886 (0.790–4.503)	0.153
CR				
Nonrupture				
Rupture	1.905 (0.642–5.654)	0.245		
Malignant potential				
Low aggressiveness				
High aggressiveness	1.217 (0.412–3.598)	0.723		
Pathological stage				
≤ I stage				
> II stage	14.250 (3.065–66.252)	**0.001**	14.727 (3.132–69.253)	**0.001**
Surgical approach				
OPN				
MIPN	0.799 (0.311–2.051)	0.640		

### Risk factors of intraoperative CR

The percentage of Bosniak IV in CR group (12.5%) was significantly lower than that in nonCR group (46.3%) (*P* < 0.001). Three pathological types including clear cell renal cell carcinoma (ccRCC), type II papillary renal cell carcinoma (pRCC), and renal carcinosarcoma were classified as high aggressiveness, and other pathological types were classified as low aggressiveness. The percentage of tumors with high aggressiveness in CR group (56.3%) was significantly lower than that in nonCR group (81.3%) (*P* < 0.001). In univariable analysis, tumor size and Bosniak classification were associated with the risk of CR. Multivariable logistic regression analysis showed that tumor size (*OR* = 1.395; 95% *CI* = 1.066–1.825; *P* = 0.015) was an independent risk factor, yet Bosniak III (*OR* = 0.342; 95% = 0.134–0.871; *P* = 0.025) and Bosniak IV (*OR* = 0.065; 95% *CI* = 0.018–0.239; *P* < 0.001) were protective factors for CR (Table 6).

**Table 6:** The logistic regression analysis for risk factors of CR

Variables	Univariate analysis		Multivariate analysis	
	OR (95% *CI*)	*p*-value	OR (95% *CI*)	*p*-value
Age	1.011 (0.984–1.040)	0.423		
BMI	0.941 (0.844–1.049)	0.272		
Hypertension	0.828 (0.372–1.844)	0.645		
Diabetes	1.933 (0.827–4.522)	0.128		
RENAL score	1.036 (0.834–1.287)	0.751		
Tumor size	1.296 (1.011–1.662)	**0.041**	1.395 (1.066–1.825)	**0.015**
Gender				
Male	Ref.			
Female	1.316 (0.602–2.879)	0.491		
Tumor location				
Left kidney	Ref.			
Right kidney	1.849 (0.867–3.942)	0.112		
Bosniak				
IIF	Ref.		Ref.	
III	0.388 (0.155–0.968)	**0.042**	0.342 (0.134–0.871)	**0.025**
IV	0.078 (0.022–0.277)	**< 0.001**	0.065 (0.018–0.239)	**< 0.001**
Surgical approach				
OPN	Ref.			
MIPN	0.535 (0.241–1.188)	0.124		

### Associations between Bosniak classification and tumor aggressiveness

All 406 cases with PN and 17 cases with CD including 7 multilocular cystic renal neoplasm of low malignant potential (MCRNLMP) and 10 ccRCC with 6 Fuhrman I and 4 Fuhrman II were integrated into the analysis which is shown in Table 7. CRM with Bosniak IV had a higher rate of ccRCC (81.4% vs 76.8% vs 36.5%), lower rate of MCRNLMP (4.0% vs 10.3% vs 28.8%), and pRCC (4.5% vs 6.2% vs 21.2%) compared to CRM with Bosniak III and Bosniak I-IIF (*P* < 0.001). CRM with Bosniak IV had a higher rate of highly aggressive tumors (84.7% vs 78.9% vs 55.8%), including ccRCC, type II pRCC, and renal carcinosarcoma, than CRM with Bosniak III and Bosniak I-IIF (*P* < 0.001). Besides, 66.7% CRM with Bosniak I-IIF were Fuhrman I grade, and 11.2% CRM with Bosniak III and 7.4% CRM with Bosniak IV were Fuhrman I grade (*P* < 0.001).

**Table 7:** Comparison of inherent aggressiveness of different Bosniak classifications

Variables	Bosniak I, II, and IIF	Bosniak III	Bosniak IV	*P*
No. of patients	52	194	177	
No. of histologic subtyp (%)				**< 0.001**
ccRCC	19 (36.5)	149 (76.8)	144 (81.4)	
MCRNLMP	15 (28.8)	20 (10.3)	7 (4.0)	
pRCC	11 (21.2)	12 (6.2)	8 (4.5)	
cRCC	1 (1.9)	7 (3.6)	6 (3.4)	
Others	6 (11.5)	6 (3.1)	12 (6.8)	
No. of malignant potential (%)				**< 0.001**
High aggressiveness	29 (55.8)	153 (78.9)	150 (84.7)	
Low aggressiveness	23 (44.2)	41 (21.1)	27 (15.3)	
No. of Fuhrman grade (%)				**< 0.001**
I	20 (66.7)	19 (11.2)	12 (7.4)	
II	10 (33.3)	142 (83.5)	139 (85.8)	
III	0	9 (5.3)	11 (6.8)	

### Effect of Bosniak classification on recurrence risk of intraoperative CR

In CR group, 2 of 4 cases with recurrence were Bosniak IV tumors, accounting for 50% (2/4) of all Bosniak IV cases, and another 2 cases were Bosniak III masses accounting for 10% (2/20). All 8 cases with Bosniak IIF did not experience recurrence. Moreover, in CD group, 2 cases with Bosniak I and 15 cases with Bosniak II did not experience recurrence with a median follow-up time of 86 months (range 13 to 139). The recurrence rate of Bosniak IV masses (50%, 2/4) was significantly higher than that of Bosniak I-III (4.4%, 2/45) (*P* = 0.029). In nonCR group, the recurrence rate of CRM with Bosniak IV (1.7%, 3/173) was comparable to that of CRM with Bosniak IIF-III (1.0%, 2/201) (*P* = 0.666).

## Discussion

When CRM rupture occurs during PN, surgeons are disturbed by the theoretical risk of tumor recurrence [11–13]. Spaliviero M. et al. [17] particularly emphasized that extreme caution and skilled laparoscopic techniques must be exercised to avoid CR and local spillage. A new technique to minimize the risk of accidental intraoperative rupture of CRM by using a SAND balloon catheter was developed by Nozaki T. et al. [18]. However, the innovative finding recently reported by Pradere B. et al. [14] proposed that intraoperative CR at PN of CRM did not increase the risk of recurrence. Although encouraging, this conclusion is less convincing for some reasons. First of all, 38 malignancy ruptures out of 50 CR from 8 institutions were enrolled into the study. The involved surgeons with different surgical experience and managements after intraoperative CR might affect oncologic outcomes. Secondly, 25% CRM were benign, which might lead to selection bias due to insignificance of benign CRM rupture. Besides, different pathological types and Fuhrman grades were not further stratified in patients with and without CR considering that tumors in CR group may have a lower malignant potential compared with nonCR group and might not be aggressive enough to lead to disease recurrence. Lastly, the shorter follow-up time of CR group than that of 9 recurrence cases might miss the later recurrence in CR group. Therefore, the larger population-based study with 406 patients pathologically diagnosed as malignant CRM in our institution was conducted to externally discover the relationship between intraoperative CR and tumor recurrence.

In our study, the incidence of intraoperative malignant CR was 7.9%, which was lower than the previous report [14]. The following reasons may explain the lower incident in our institution. Firstly, our data came from a large volume center, and all the cases were performed by experienced surgeons. Secondly, only malignant CRM were enrolled into our study, and the benign CRM probably ruptured more easily. Lastly, some cases of CR might be incorrectly classified as unruptured because surgeons might not describe CR in surgery records.

Our study found that the risk of recurrence in patients with CR was higher than that in patients without CR. This is consistent with the theoretically increased recurrence risk due to tumor spillage [11–13]. Compared with cases without recurrence in CR group, the pathological type of cases with recurrence is more aggressive. Two of 3 cases with type II pRCC experienced recurrence. On the contrast, 2 cases with type I pRCC did not experience recurrence. This is consistent with the fact that in pRCC, type II is more aggressive than type I [19–21]. Renal carcinosarcoma is an extremely rare tumor that progresses rapidly and has a poor prognosis [22, 23]. In our study, one patient with renal carcinosarcoma immediately suffered from local recurrence and distance metastasis within 2 months after CR. Mixed epithelial and stromal tumors (MEST) tend to be benign. However, some studies reported the presence of malignant MEST [24–27]. In our study, one patient with malignant MEST experienced recurrence at 23 months after CR. Yap Y. S. et al. [28] also reported that the intraoperative CR probably was an important risk factor for recurrence in MEST cases. Although ccRCC is considerably aggressive, no recurrence occurred in all 14 cases with ccRCC in our study which may be due to the low Fuhrman II or I grade in these cases. Besides, all 6 cases with MCRNLMP did not experience recurrence due to the low malignant potential. Moreover, in CD group, 10 ccRCC with 6 and 4 Fuhrman I and II, respectively, and 7 MCRNLMP cases also did not experience recurrence. Therefore, the conclusion that intraoperative CR had negative impacts on oncologic outcomes was far from convincing. The CR of tumors with low malignant potential perhaps have no negative impact on the prognosis. Once an extremely aggressive tumor ruptures, it can bring catastrophic consequences for the patients. However, the exact pathological type was not known until a few days after surgery. It is vitally important to preoperatively identify cases with high risk of recurrence after CR.

Bosniak classification [29, 30] is a classical system which categorizes CRM into five groups of different malignancy risks on the basis of computerized tomography findings. A multicenter study [31] showed that CRM with Bosniak IV had a higher malignant potential than CRM with Bosniak III. In our study, CRM with Bosniak IV had a significantly higher rate of ccRCC and lower rate of MCRNLMP and pRCC compared to CRM with Bosniak III and Bosniak I-IIF. After three pathological types including ccRCC, type II pRCC, and renal carcinosarcoma were classified as highly aggressive tumors and other pathological types were classified as less aggressive tumors, CRM with Bosniak IV had a higher rate of highly aggressive tumors than CRM with Bosniak III and Bosniak I-IIF. Besides, CRM with Bosniak IV also had a significantly higher rate of Fuhrman II and III grade than CRM with Bosniak III and Bosniak I-IIF. In summary, CRM with Bosniak IV were more aggressive than CRM with Bosniak I-III. In this study, 2 of 4 cases with Bosniak IV and 2 of 20 cases with Bosniak III experienced recurrence. Meanwhile, no recurrence occurred in 8 CR cases with Bosniak IIF and 17 CD cases with Bosniak I or II. The recurrence rate of CRM with Bosniak IV (50%) was significantly higher than that of CRM with Bosniak I-III (4.4%). Moreover, in nonCR group, the recurrence rate of CRM with Bosniak IV was comparable to that of CRM with Bosniak IIF-III. Therefore, for CRM with higher Bosniak classification, especially Bosniak IV, rupture should be avoided because of the higher risk for recurrence.

Another important finding in our study was that tumor size and Bosniak classification were independent risk factors for CR. It is well understood that the larger the diameter of CRM, the greater the possibility of rupture during surgery. The cyst wall of CRM with Bosniak IV is thicker than that of CRM with Bosniak IIF and III [29], which may contribute to the higher probability of rupture in CRM with low Bosniak staging and explain the earlier recurrence in nonCR cohort than the CR group for the malignancy nature in Bosniak IV CRM. The larger number of CRM rupture with Bosniak IIF-III led to the larger number of less aggressive tumors in CR group, which could weaken and even cover up the true effect of intraoperative CR on oncologic outcomes.

The major limitation of our study is the retrospective and single-centered nature. Besides, the surgery records lack reliability for that some CR cases might be incorrectly classified as unruptured. Moreover, different techniques of various surgeons could lead to bias, and the follow-up time was not long enough for more convincing results. Prospective multicenter studies with a larger number of patients and longer follow-up time are expected in the future to further reassure the conclusions.

## Conclusions

Our study showed that intraoperative CR of malignant CRM indeed had negative impacts on oncologic outcomes. CRMs with Bosniak IV staging were more aggressive and therefore had a higher risk of recurrence after CR than CRMs with Bosniak I-III staging. However, Bosniak IV CRM had a lower risk of CR than CRM with Bosniak I-III, which could weaken and even cover up the true effect of intraoperative CR on oncologic outcomes. Urologists should still give enough attention to avoid CR, especially CRM with Bosniak IV.

## Data Availability

The datasets are available from the corresponding author on reasonable request.

## Abbreviations

CRM: Cystic renal masses
PN: Partial nephrectomy
CR: Cyst rupture
CD: Cyst decortication
MIPN: Minimally invasive partial nephrectomy
RAPN: Robot-assisted partial nephrectomy
RFS: Recurrence-free survival
MFS: Metastasis-free survival
CSS: Cancer-specific survival
OS: Overall survival
ccRCC: Clear cell renal cell carcinoma
pRCC: Papillary renal cell carcinoma
MCRNLMP: Multilocular cystic renal neoplasm of low malignant potential
MEST: Mixed epithelial and stromal tumors
